# Microstructural Evolution of Poly(ε-Caprolactone), Its Immiscible Blend, and In Situ Generated Nanocomposites

**DOI:** 10.3390/polym12112587

**Published:** 2020-11-04

**Authors:** Iurii Vozniak, Ramin Hosseinnezhad, Jerzy Morawiec, Andrzej Galeski

**Affiliations:** Centre of Molecular and Macromolecular Studies, Polish Academy of Sciences, 90363 Lodz, Poland; wozniak@cbmm.lodz.pl (I.V.); jemor@cbmm.lodz.pl (J.M.)

**Keywords:** nanocomposite, shear-induced crystallization, immiscible polymers, PCL, lamellae fragmentation

## Abstract

Polymer–polymer systems with special phase morphology were prepared, leading to an exceptional combination of strength, modulus, and ductility. Two immiscible polymers: poly(ε-caprolactone) (PCL) and polyhydroxyalkanoate (PHA) were used as components for manufacturing a nanoblend and a nanocomposite characterized by nanodroplet-matrix and nanofibril-matrix morphologies, respectively. Nanofibrils were formed by high shear of nanodroplets at sufficiently low temperature to stabilize their fibrillar shape by shear-induced crystallization. The effects of nanodroplet vs. nanofiber morphology on the tensile mechanical behavior of the nanocomposites were elucidated with the help of in situ 2D small-angle X-ray scattering, microcalorimetry and 2D wide-angle X-ray diffraction. For neat PCL and a PCL/PHA blend, the evolution of the structure under uniaxial tension was accompanied by extensive fragmentation of crystalline lamellae with the onset at strain e = 0.1. Limited lamellae fragmentation in the PCL/PHA composite occurred continuously over a wide range of deformations (e = 0.1–1.1) and facilitated plastic flow of the composite and was associated with the presence of a PHA nanofiber network that transferred local stress to the PCL lamellae, enforcing their local deformation. The PHA nanofibers acted as crystallization nuclei for PCL during their strain-induced melting–recrystallization.

## 1. Introduction

Poly(ε-caprolactone) (PCL) is one of the most versatile thermoplastic polymers with good mechanical properties, biocompatibility, non-toxicity vs. biological tissues, and biodegradability in several biotic environments. Due to the highest stability of its crystal structure and lack of polymorphism upon tensile/compression deformation or thermal treatment, PCL is admitted as a model semicrystalline polymer, an alternative to polyethylene or polypropylene. Over the past decades, the structural formation of PCL under different conditions has attracted much attention [1,2,3]. Moreover, various attempts, such as copolymerization, blending, and reinforcement with nanofillers and natural fibers, have been undertaken to improve the tensile strength of PCL [4,5,6,7,8,9,10,11,12,13]. Regulation of the hierarchical structure of PCL via shear stress-induced orientation, which leads to the formation of a shish-kebab-like structure, have also been shown to be an effective way to improve its strength and rigidity [14]. Finally, in recent years, experimental evidence supports the idea that the morphology of the minor phase can pay a critical role in the improvement of the mechanical properties of PCL, leading to an exceptional combination of strength, modulus, and ductility. This was achieved by transformation of polymer blend into polymer–polymer composites [15,16,17]. However, it is still unclear what mechanism of plastic deformation underlies the improvement of the plasticity of such systems. A small number of structural investigations have been carried out to explain the relationship among the PCL microstructure and its properties [10,18]. At the same time, since the deformation process is strongly dependent on the nanoscale morphology, which is constituted by both crystalline lamellae and the amorphous layers which are highly interconnected, various descriptions of the possible mechanisms of plastic deformation of PCL have been addressed [19,20]. For instance, Park et al. [19] used a strain-induced melting–recrystallization mechanism (mechanical melting) to describe the process of the plastic deformation of neat PCL in a tensile deformation mode. It was found that in the yielding region, fragmentation of the chain-folded lamellae into a checkerboard-like arrangement with subsequent melting of the chain-folded lamellae and recrystallization into chain-extended lamella took place. In the plastic deformation region, chain-extended lamellas further developed simultaneously with a high degree of orientation of PCL crystals. Men et al. [20] revealed the role of mechanism of cavitation in the process of the plastic deformation of neat PCL and its miscible blends with poly(vinylmethylether) (PVME) and poly(styrene-co-acrylonitrile) (SAN) under uniaxial stretching. It appeared that addition of amorphous SAN determines a significant reduction in the entanglement density of the amorphous phase of PCL, whereas an introduction of PVME does not affect it. As a result, stretching did not initiate cavitation in the PCL/SAN blend and resulted only in a rearrangement of lamellae stacks along the drawing direction forming the fibril structure, while neat PCL and PCL/PVME initiated cavitation. The authors associated this phenomenon with the fact that the cavities form only in crystalline lamellae with the orientation parallel to the stretching direction and then grow passing through several interlamellar amorphous layers.

In recent years, much experimental evidence supports that the mechanism of plastic deformation of polymer matrix depends to a large extent on the morphology of the minor polymer phase. Past studies of polymer–polymer systems demonstrated that designed special phase morphologies, such as entangled nanofibers of one polymer inside the matrix of another polymer or hybrid shish-kebab-like structures, can lead to an exceptional combination of strength, modulus, and ductility [21,22,23]. Therefore, the understanding of the mechanism of plastic deformation responsible for the improvement of plasticity of such systems as well as their structural changes during tensile deformation including fracture are of importance.

This article is the first to consider the role of the fragmentation of crystal lamellae in the ability of polymer–polymer system with fibril/matrix morphology to undergo significantly greater plastic deformation as compared to droplet/matrix morphology. In this article, The PCL matrix and the dispersed polymer phase based on polyhydroxyalkanoate (PHA) were chosen as components of the polymer–polymer system. The possibility of simultaneously improving the strength and ductility of PCL matrix by converting a PCL-based blend to in situ generated nanocomposite via morphology modification in the minor polymer phase from droplets to fibers was also demonstrated. A specific feature of in situ generated nanocomposites lies in the fact that the crystallization of the minor polymer phase (i.e., PHA) is forced by the high shear rate [22], and its crystals are crystallized at a temperature higher than the softening temperature of the PCL matrix. Moreover, effective fibrillation of the molten droplets of the dispersed polymer can also be achieved at the same shear rate and temperature [24]. A slit die extrusion process is proposed for achieving a high shear rate and elongation at relatively low temperature and also for increasing the residence time in the extruder and facilitating the formation of the nano-fibril network at low concentrations of PHA. Such an arrangement allows for shear-induced crystallization of highly deformed inclusions of PHA inside the extruder and inside the slot capillary.

The influence of the morphology of the dispersed polymer phase (droplet/fibers) on the specific feature of structural evolution of PCL/PHA system during the tensile deformation was studied by means of in situ 2D small-angle X-ray scattering (SAXS), DSC, and 2D WAXS techniques.

## 2. Materials and Methods

### 2.1. Materials

Commercial-grade semi-crystalline hydrophobic biodegradable polycaprolactone (PCL) with Mn 80,000 g/mole was purchased from Sigma-Aldrich (Darmstadt, Germany) in the form of pellets (~3 mm). This grade of PCL, with a melting point at around 60 °C and density of 1.145 g/mL at 25 °C, was used as the matrix for preparation of composites.

Polyhydroxyalkanoate (PHA) with the trade name Mirel P5001 from Metabolix (Cambridge, MA, USA) was chosen in order to reinforce the PCL. The PHA was produced by fermentation of sugar. It was a crystalline polymer and showed a melting point at 170 °C and a density of 1.30 g/cm^3^. It had a high melt strength suitable for blown and cast film extrusion. The P5001 is heat sealable and has excellent tensile properties. The PHA biodegrades in fresh or saltwater marine environments.

### 2.2. Sample Preparation

The PCL/PHA blend and in situ generated PCL/PHA nanocomposite were prepared (both components were dried for 8 h at 60 °C) following the procedure described earlier [25]. Temperature zones of a twin-screw extruder were set increasingly from 160 to 190 °C, while this temperature gradient descended for a single-screw extruder from 175 °C (feed section) to 135 °C (slit die). During melt blending of 6 wt% of PHA with PCL a 0.2 wt% of Irganox 1010 (BASF, Poland) for prevention of the thermooxidative degradation and 0.2 wt% of metal deactivator Irganox MD 1024U (BASF, Poland) for additional deactivation of catalysts were added. The concentration of the minor polymer phase was chosen at 6 wt%, because it corresponded to the minimum concentration at which the formation of a continuous network of fibrils occurs [26].

### 2.3. Mechanical and Thermal Properties

The tensile properties of neat PCL, its blend, and in situ generated composite were measured in Instron-5582 (Universal Testing Machine) at a strain rate of 5%/min according to ISO 527-2. Strain measurement was performed by use of a clip-on extensometer. Toughness was determined by integrating the stress–strain curve. Seven specimens were tested for each blend and composite at room temperature. Melting and crystallization kinetics were probed with DSC Q20 (TA Instruments, New Castle, DE, USA) under nitrogen purging condition (20 mL/min). The measurements were carried out by heating and subsequent cooling of samples with the rate of 10 °C/min. In order to perform in situ DSC tests, the specimens were cut out from stretched samples. In this regard, immediately after reaching the specific strains during the tensile test, the deformed samples were fixed by applying a dense polystyrene solution prior to unloading. The solution was prepared by dissolving polystyrene in toluene. The deformed samples were released after solidification of polystyrene. The melting thermograms were recorded at the heating rate of 10 °C/min under nitrogen flow.

Thermal gravimetric analysis (TGA) experiments were conducted with a TA Instruments Hi-Res TGA 2950 Thermogravimetric Analyzer (New Castle, DE, USA) at the heating rate of 10 °C/min in nitrogen. It was used to thermally decompose milligram samples under controlled heating and environmental conditions in nitrogen to detect their thermal stability and weight reduction. Two types of plot were available as a result. A plot of specimen weighed against temperature (TGA curve) provided thermal decomposition temperatures with the residue amount as a function of temperature. The second plot, a derivative of the TGA curve, indicated mass loss rate depending on an increase in temperature.

### 2.4. Scanning Electronic Microscopy (SEM)

The morphology of samples, cryogenically fractured along the extrusion direction and coated with gold, was investigated with a JEOL JSM-5500 LV (Tokyo, Japan) scanning electron microscope.

### 2.5. Rheological Measurements

The rheological behavior of the materials was examined using a strain-controlled rotational rheometer ARES LS2, TA Instruments (New Castle, DE, USA). Uniaxial extension tests of molten samples were performed using extensional viscosity fixture, EVF, by TA Instruments, (New Castle, DE, USA) attached to the ARES rheometer. The 18 × 10 × 0.7 mm^3^ rectangular specimens were prepared by hot compression molding at 200 °C in the standard mold, provided also by TA Instruments with the EVF or were cut out from extruded tapes. The specimens were uniaxially extended at 60 °C with a constant Hencky strain rate, $\dot{\varepsilon }$, of 0.01, 0.05 or 0.1 s^−1^. The temperature was set in a way that the elastic force of the minor phase would be sufficient to overcome the plastic flow of molten matrix. The effect of nanofibers would be concealed at higher temperatures due to the dominant plasticity of the major phase. The tensile stress growth coefficient, $\eta_{E}^{+}$η(t,$\dot{\varepsilon }$), as a function of time, t, at a given Hencky strain rate, $\dot{\varepsilon }$, was measured. The tests for each material were repeated at least eight times, and the results were averaged.

### 2.6. In Situ 2D SAXS

The lamellar structure of studied materials and its variation with strain was probed with two-dimensional small-angle X-ray scattering. The test was facilitated by employing a home-assembled in situ SAXS apparatus where a Linkam MicroTester, TST350, (Tadwarth, UK) was connected to GeniX Cu-LD (Xenocs SAXS Instrument, Sassenage, France). Online stress–strain curves enabled precise stretching of dumbbell-shaped samples (l = 10 mm, w = 3 mm, and th = 0.5 mm) at a strain rate of 1% min^−1^. At specific strains, stretching was paused shortly to capture high-quality patterns with minimum inevitable stress relaxation during the acquisition. However, the acquisition time was 1 min, which corresponded to a strain of 1%.

### 2.7. 2D WAXS

Two-Dimensional Wide-Angle X-ray Diffraction, (2D WAXS), images were registered with flat X-ray camera equipped with imaging plates Fujifilm (Tokyo, Japan) and coupled to Cu Kα source (sealed tube operating at 30 kV and 50 mA, by Philips (Koninklijke, The Netherlands).

## 3. Results

### 3.1. Characterization of PCL, PCL/PHA Blend and Composite

**Figure 1:**
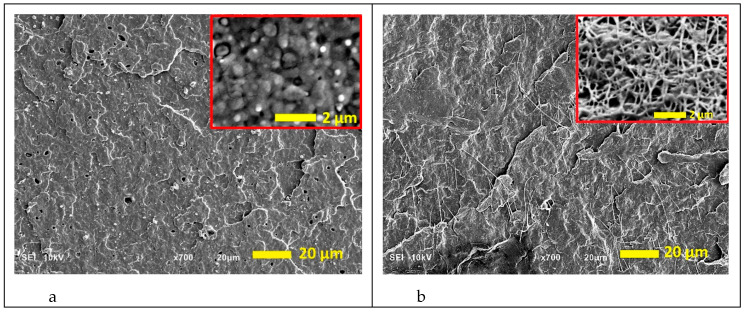
SEM micrographs of the cryo-fractured surfaces of the poly(ε-caprolactone) (PCL)/polyhydroxyalkanoate (PHA) blend (**a**) and in situ generated nanocomposite (**b**). PHA spherical inclusions and nanofibers are presented in the insets at higher magnification, details were exposed through mild etching of the fractured surfaces with chloroform to remove PCL as per Wu et al. [27].

Figure 1 presents the morphology of the cryogenically fractured surfaces of the PCL/PHA blend and in situ generated PCL/PHA nanocomposite with 6 wt% of PHA. It can be seen that the PHA phase exhibits droplet-matrix (Figure 1a) or fibril-matrix (Figure 1b) morphologies for the blend and composite, respectively. In the case of a blend, nano-sized PHA inclusions were uniformly distributed in the PCL matrix. Most of PHA was dispersed as spherical particles having a size in the range of 400–850 nm, and the averaged size was approximately 520 nm. That facilitated effective homogeneous distribution of PHA fibers formed by in situ fibrillation. The PHA fibrils had diameters ranging from 70 to 100 nm, and their aspect ratios were at least 100. Thus, the PHA nanofibril-reinforced PCL nanocomposite was successfully fabricated using an in situ fibrillation method. The PHA nanofibers could also induce plastic deformation of the PCL matrix during cryogenic fracture, which led to the observed PCL fibers with a diameter of 100 to 450 nm. Moreover, PHA nanofibers form the physical network structure, as evidenced by the viscoelastic behavior of the neat PCL, PCL/PHA blend and in situ generated nanocomposite (shown in Supplementary Materials).

**Figure 2:**
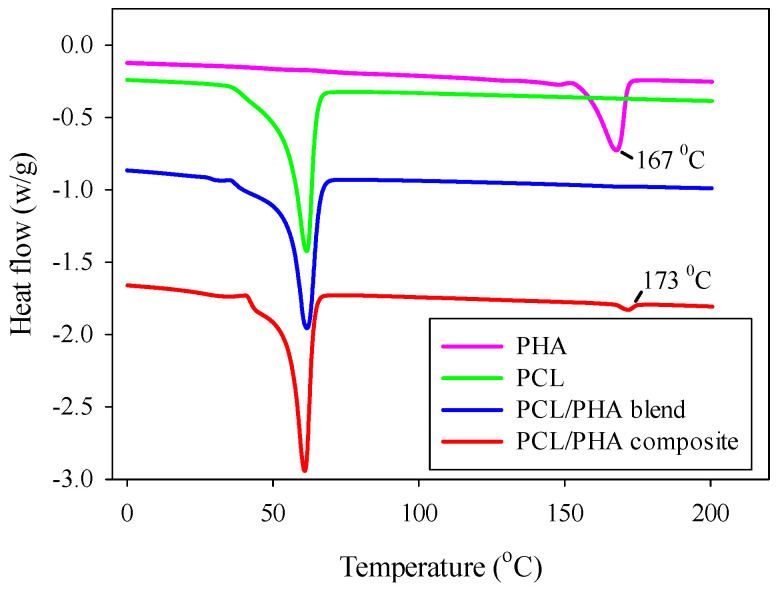
Melting endotherms of neat PHA, PCL, PCL/PHA blend and in situ generated nanocomposite. Curves for PCL/PHA blend and for PCL/PHA composite were shifted vertically for better visualization.

Formation of the fibrillar structure also contributed to the creation of PHA crystals with a higher melting temperature (Figure 2). Moreover, in the case of in situ generated nanocomposites, a sharper (distinct) melting peak for PHA was observed in comparison with the blend. The melting temperature and crystallinity degree of PCL matrix did not change and were 61 °C and of approximately 45%, respectively, for both systems.

The DSC results correlate with the results of TGA (shown in Supplementary Materials), which demonstrate higher thermal degradation temperatures in the case of PCL/PLA composite compared to the PCL/PLA blend.

**Figure 3:**
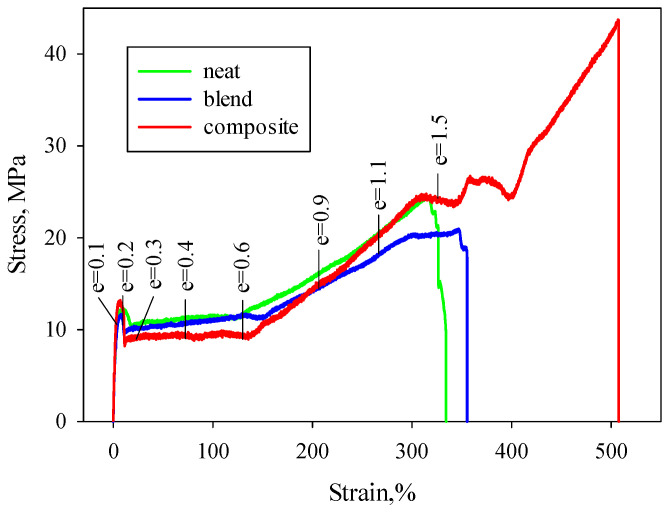
Stress–strain curves for neat PCL, PCL/PHA blend, and in situ generated nanocomposite.

The presence of a PHA nanofibril network caused a change in the mechanical behavior of PCL polymer matrix, leading to an exceptional combination of its strength, modulus, and ductility. Exemplary stress–strain (S–S) dependencies determined during uniaxial tensile test on neat PCL, PCL/PHA blend and in situ generated composite are collected in Figure 3, whereas the mechanical data (average values) are detailed in Table 1.

Adding 6 wt% of the PHA into PCL results in a minor decrease in Young modulus from 330 to 306 MPa as well as yield stress and stress at break from 12.0 and 24.0 MPa to 11.4 and 20.5 MPa, correspondingly. At the same time, strain at break and toughness of the PCL/PHA blend are higher than that of the neat PCL (357% and 42.4% vs. 334% and 40.4%). In the case of the in situ generated PCL/PHA composite characterized by fibrillar morphology, the Young modulus increased to 690 MPa, stress at break-up to 43.7 MPa, and strain at break reached 508%. Toughness doubled. Thus, for PCL/PHA composites, a simultaneous increase in strength, stiffness, and ductility was observed compared to neat PCL.

The increase in strength and modulus associated with the formation of the nanofibril network is discussed in References [22,23] and was due to the extensive load-bearing capacity of the long polymer nanofibrils forming the network as well as the existence of larger interfacial areas between the polymer nanofibers and the polymer matrix. At the same time, a 1.5-fold increase in strain at break as well as two-fold increase in toughness when creating a polymer–polymer composite in the case of a ductile polymer matrix, such as PCL, requires more detailed consideration.

Since the characteristics of the S–S curves should correlate to the structural changes, the in situ 2D SAXS, DSC, and 2D WAXS tests were performed. The characteristic points were selected on the S–S curves, which corresponded to the yield region: before and after the yield point (the respective true strain e = 0.1 and e = 0.2), plastic deformation region (e = 0.3; 0.4; 0.6), and the strain hardening region (e = 0.9; 1.1; 1.5). For all characteristic points, the corresponding qualitative description of the 2D SAXS, DSC, and 2D WAXS data were collected.

### 3.2. In Situ SAXS Results

**Figure 4:**
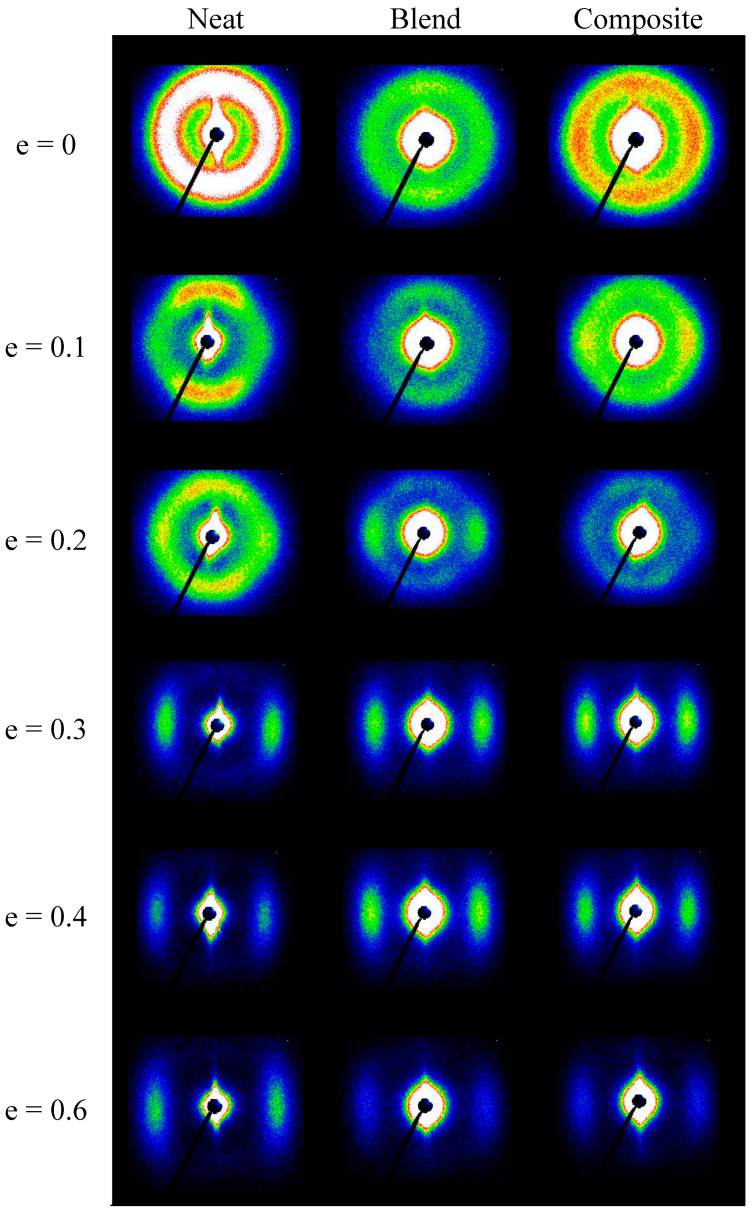

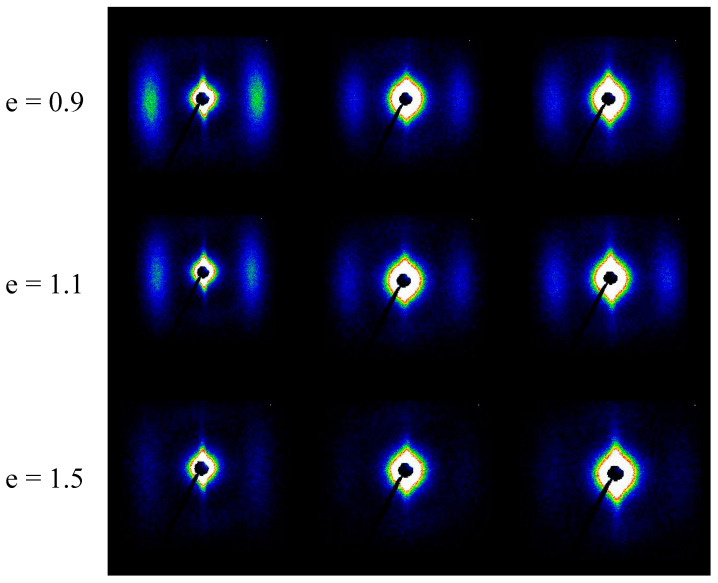
The 2D SAXS patterns of neat PCL, PCL/PHA blend, and composite samples recorded at different elongation. The drawing direction is horizontal.

Figure 4 shows the representative 2D SAXS patterns of neat PCL, PCL/PHA blend and composite. Before deformation (e = 0), the SAXS patterns form uniform rings, indicating the absence of any preferred crystals orientation in the undeformed materials. At the beginning of yielding region (e = 0.1), the SAXS patterns grew oblate and signs of four-point pattern appeared. In the ending of yielding region, the SAXS patterns started to transform from oblate to a four-point pattern and then to a six-point pattern (e = 0.2, when stress in S–S curves began to decrease), which represents an overlapping of the four-point pattern with the two-point one. According to Reference [19] for PCL, the presence of a six-point SAXS pattern is associated with the coexistence of two types of crystalline lamellas: chain-folded lamellae (leading to four-point pattern) and new formed chain-extended lamellae (adding two-point pattern). It is noted that with increasing strain, the proportion of chain-folded lamellae decreases, and the proportion of chain-extended lamellae correspondingly increases due to the destruction of the former. This transition is caused by fragmentation of the lamellae followed by the escaping of the chains from the initial chain-folded lamellae into amorphous region, their elongation and subsequent crystallization into new chain-extended lamellae oriented along the stretching direction. At the beginning of plastic deformation region (e = 0.3) the four-point pattern disappeared completely, and only two-point pattern remained for all types of tested samples. Further elongation leads to a decrease in the intensity of the two-point pattern.

**Figure 5:**
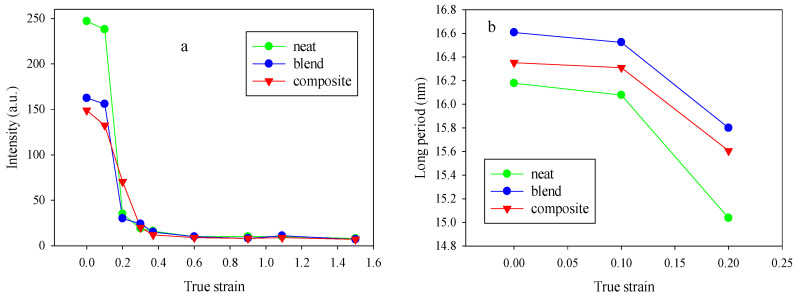
Dependencies of the maximum intensity (**a**) and long period (**b**) in the four-point patterns for the neat PCL, PCL/PHA blend and composite samples.

Figure 5 compares intensities observed at maxima in the four-point pattern for PCL, PLC/PHA blend and composite samples stretched to various true strains. It was observed that for the neat PCL and the PCL/PHA blend the maximum intensity initially did not change significantly with strain up to approximately e = 0.1 but then began to decrease progressively with further advance of the strain. For the PCL/PHA, composite crossover point seen near e = 0.1, but the decline in intensity occurs more smoothly, it is not as sharp as in the case of the neat PCL or PCL/PHA blend.

In general, for semicrystalline polymers, the intensity reflects the population of lamellae that are rotated to the specific tilt and contributed to the maxima in intensity of the scattering, due to operation of the active deformation mechanisms (interlamellar shear supported by crystallographic slip systems) [28]. The maximum intensities in the four-point patterns begin to decrease steeply at strains above e = 0.1 for the PCL and PCL/PHA blend samples. This character of the dependence of the intensity on strain (the presence of a crossover point) indicates that the decrease in intensity is not associated with the change of lamellae orientation, but with a reduction in the global population of lamellae, probably due to the lamellae damage process via their extensive fragmentation, beginning at this range of strain. At the same time for the PCL/PHA composite, a smoother change in intensity was observed. Based on the observations reported in References [28,29], it can be implied that in the latter case, there were probably two sub-processes of lamellae fragmentation: limited fragmentation active at the beginning of the plastic flow process and a more widespread fragmentation process which happened in the late stages of plastic flow.

The observed difference in the character of the process of fragmentation (extensive vs. limited) can be due to the fact that PHA nanofibers are able to transfer locally concentrated stresses to the PCL lamellae, enforcing their local deformation. After reaching a certain strain the lamella stacks with crystals perpendicular to tensile stress could undergo buckling instability which leads to cooperative kinking and then to limited fragmentation of a fraction of lamellae which oriented accordingly. As mentioned in References [28,29], the cooperative kinking of lamellae could be considered as a source of fragmentation. With increasing strain, the crystalline lamellas gradually rotate along the tensile direction and their subsequent fragmentation occurs.

Thus, buckling and subsequent kinking of lamellae could induce a fragmentation of stiff lamellae and cause subsequent easy plastic deformation for lamellae through relatively easy crystallographic slip supported by interlamellar shear. In some cases, this can lead to a shift in the beginning of the process of strain hardening towards higher strain or/and the appearance of a second yield, which was observed for the PCL/PHA composite (Figure 3).

The behavior of a long period with an increase in the strain (Figure 5b) also confirms the assumption that the process of fragmentation of crystalline lamellae proceeds. Indeed, there is a crossover point at e = 0.1 for neat PCL as well as for PCL/PHA blend and composite. It is noteworthy that the long period for the PCL/PHA blend was slightly higher than for the neat PCL and PCL/PHA composite. The latter indicates a partial incorporation of the amorphous phase of PHA between crystalline PCL lamellas.

**Figure 6:**
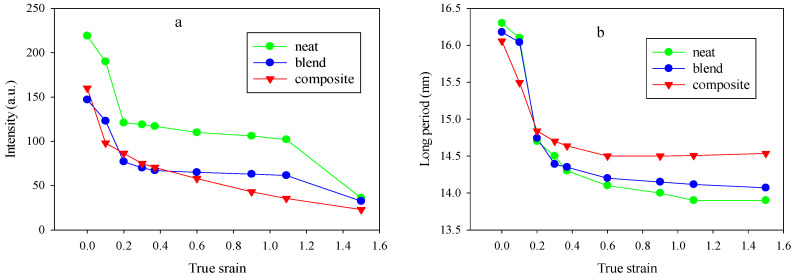
Dependencies of the maximum intensity (**a**) and long period (**b**) in the two-point patterns for neat the PCL, PCL/PHA blend and composite samples.

Figure 6 shows the dependencies of the maximum intensity (a) and long period (b) in the two-point patterns for the PCL, PCL/PHA blend, and composite samples stretched to various strains.

Higher values of initial intensities may be due to the fact that below e = 0.2 for neat the PCL, PCL/PHA blend and composite, the four-point patterns were not fully oriented due to the low lamellae orientation so they could contribute to the intensity of the two-point pattern. At e > 0.3, the two-point pattern was completely separated from the four-point pattern contribution.

A comparison of Figure 5a and Figure 6a shows that the intensity of scattering contributing to the new long period (a two-point SAXS pattern) was lower compared to the scattering by the parent structure (four-point SAXS pattern) either at moderate or even at high strain, which indicates that lamellar ordering of the parent structure was destroyed significantly by the fragmentation process. It can be assumed, that after fragmentation crystalline lamellas become less constrained and can easily undergo rotations. Such lattice restructuration in order to reduce the interface energy via interlamellar and interlamellar slip mechanisms could ultimately lead to a fibrillar structure formation. This suggestion also originated from the fact that both phases: crystalline and amorphous become oriented during stretching. At large strain, they form a highly oriented fibrillar structure [30,31,32]. According to Strobl [33], in the case of polyethylene there is a critical true strain of about 0.6 for the onset of fibril formation that is irrespective of crystallinity, deformation temperature, strain rate, and lamellar thickness. The SAXS patterns presented in Figure 4 indicate the appearance of elongated central rings at strains above true strain of 0.2 (elongation around 20% and above) that may be associated with an increase in the volume due to the formation of gaps that are formed between individual fibrils when they assume their identity. This is in contrast to classical cavitation with initiation and growth of bubbles. The latter follows from the fact that the maximum scattering intensities of the central rings in stretched samples are close to or even less than those measured for the non-stretched ones, thus it can be considered that the scattering on nanometer voids in the structure is negligible.

With increasing strain, for all studied materials, the long period for the two-point patterns gradually decreased and reached a plateau. A similar behavior of the new long period was observed in References [19,20] for neat PCL and PCL blends with miscible amorphous polymers and was explained by the implementation of separation of the fluid amorphous layers, interlamellar block slip and stress–induced fragmentation-recrystallization processes.

It should be noted that the higher absolute values of a long period occur in the case of the PCL/PHA composite. The different morphology of the dispersed PHA phase can affect the crystallization process of PCL crystallites in different ways. Apparently, the presence of PHA nanofibers leads to the formation of thicker PCL crystals, while PHA nanodroplets practically did not affect the crystallization process of PCL.

### 3.3. DSC Results

**Figure 7:**
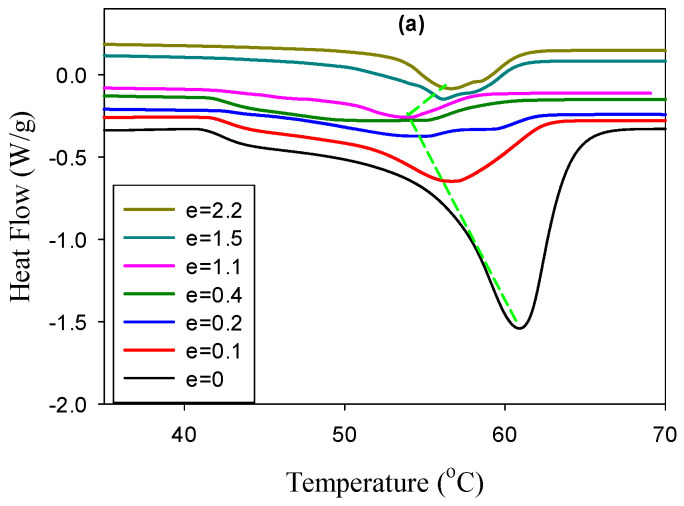

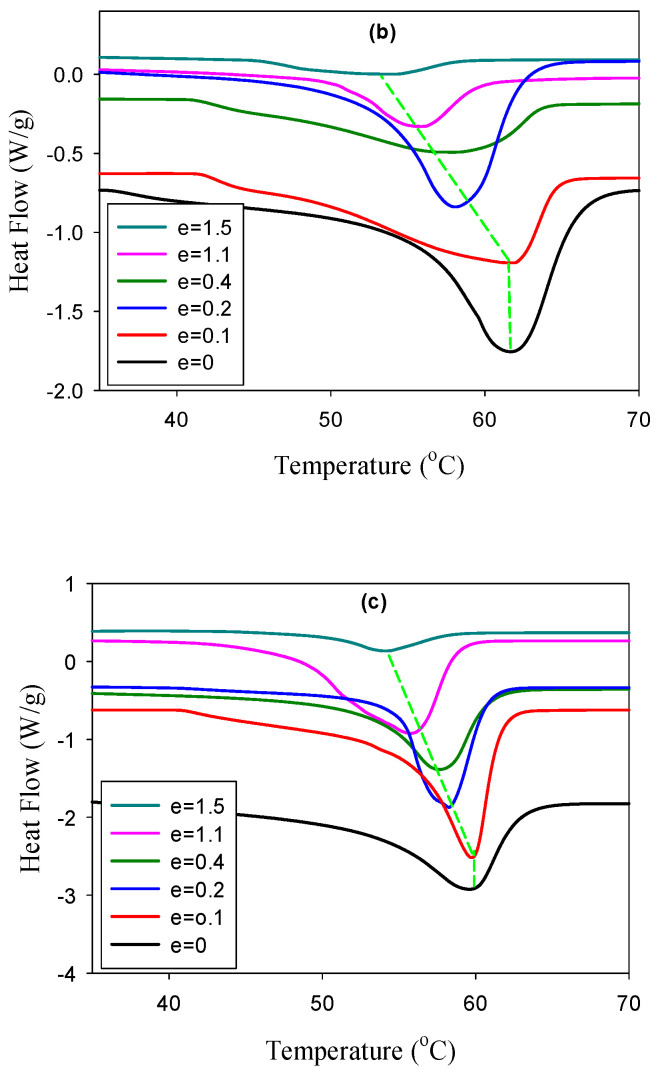
DSC melting thermograms of the deformed samples of the PCL/PHA composite (**a**), blend (**b**), and neat PCL (**c**). The true strains are indicated in the plots.

The DSC experiments were carried out to reveal the PCL crystal evolution during tensile stretching and the resultant thermograms of neat PCL, PCL/PHA blend as well as composite at different true strain are shown in Figure 7. It can be seen that for all studied samples, with increasing strain, a shift in the melting temperature towards lower temperatures is observed. The character of the melting temperature change correlates with the observed behavior of the maximum intensity and the long period (Figure 5 and Figure 6).

Indeed, in the strain range 0.1–0.2 for the neat PCL and PCL/PHA blend, a sharp decrease in the melting temperature takes place, after which it decreases monotonically with a further increase in strain. In the case of a composite, a gradual decrease in the melting temperature is observed in the strain region 0.1–1.1, which is also consistent with the observed characteristic behavior of the maximum intensity and the long period. At the same time, for strain above 0.2, a double melting peak appears for the PCL/PHA composite. It should be noted that the position of the low-temperature melting peak shifts toward higher temperatures with increasing strain, while the high-temperature peak continues to shift toward lower temperatures. It can be assumed that PHA nanofibrils act as crystallization nuclei for PCL crystals.

### 3.4. 2D WAXS Results

**Figure 8:**
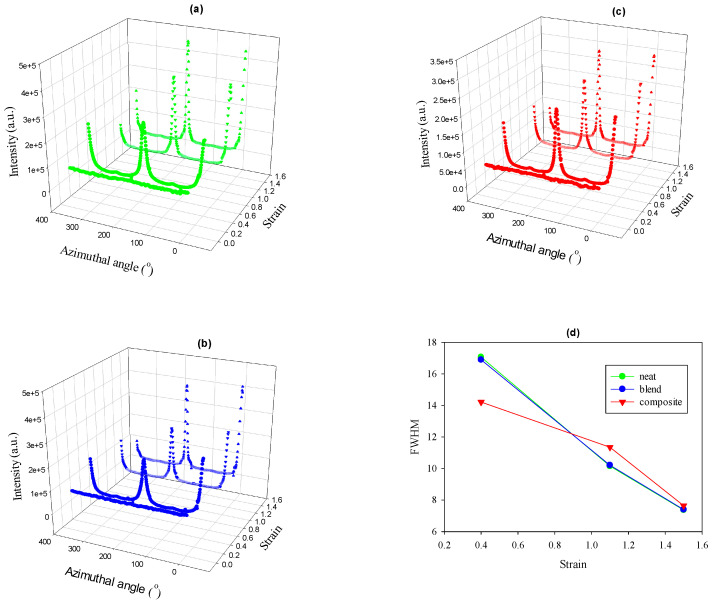
Azimuthal scans of the 110 reflection of PCL for neat PCL (**a**), PCL/PHA blend (**b**), PCL/PHA composite (**c**), and the relevant FWHMs (**d**).

Figure 8 shows the azimuthal scans of the 110 reflection as a function of true strain and full width at half-maxima (FWHM) calculated from the peak at 180°. The selected range of true strain values is 0.4–1.5; therefore, the development of the crystal orientation and the associated decrease in the FWHM are associated with crystals formed as a result of fragmentation of the initial crystalline lamellas that occurs at e = 0.1–0.2. It is seen that with increasing strain, the orientation of the crystals started to develop and FWHM decreased for all materials. However, for the composite, the decrease in the FWHM values occurred at a lower rate, which indicates a slower orientation of the PCL crystals and correlated with the SAXS results, proving the gentler lamellae reorganization in the case of the composite.

## 4. Conclusions

**Figure 9:**
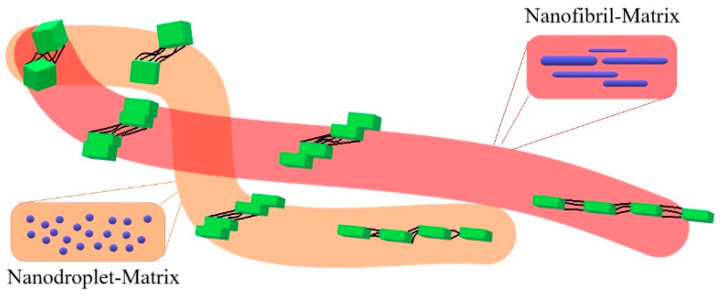
Scheme of the different character of the fragmentation process for polymer blend and polymer–polymer composite.

In this work, on an example of poly (ε-caprolactone) (PCL) and polyhydroxyalkanoate (PHA), which respectively form PCL with 6 wt% PHA blend and composite, the effect of nanodroplet/matrix and nanofibril/matrix morphologies on the performance of polymer–polymer systems was studied. It was shown that in the case of composite, the reinforcing effect was accompanied by a 1.5 fold increase in strain at break and a two-fold increase in toughness. At the same time, for blend there was a slight decrease in strength and modulus with a slight increase in strain at break and toughness. The difference in the observed reinforcing effects for composite and blend was associated with the presence of nanofibers which in contrast to nanodroplets form the effective load-bearing entanglements. The latter also cause different plastic response of the formed polymer–polymer systems. Using in situ 2D-SAXS, it was shown that in the case of the PCL/PHA blend and neat PCL, extensive fragmentation of the crystalline lamellae took place, while limited fragmentation occurs for PCL/PHA composite. The limited fragmentation causes a relatively easy plastic deformation by transferring the locally concentrated stresses through PCL lamellae. The PHA nanofibers were responsible for altering the character of PCL lamellae fragmentation process. As a result, significantly greater strain at break values were achieved in the PHA/PCL composite. The formation of thicker PCL crystals as a result of their strain-induced melting–recrystallization in the presence of PHA nanofibrils also contributed to the achievement of high ductility values for composites. At the same time, PHA nanodroplets practically did not affect the recrystallization process of PCL. The 2D WAXS results display a slower rate of the PCL crystal orientation for the PCL/PHA composite compared to neat PCL as well as PCL/PHA blend. The scheme in Figure 9 demonstrates that the mechanism of the lamellae fragmentation is the key to understanding the enhancement of plasticity.

## 5. Patents

Means of manufacturing polymer composites reinforced with polymer nanofibers. Polish Patent Application P.429273, inventors: I. Vozniak, R. Hosseinnezhad, A. Galeski.

## Figures and Tables

**Table 1:** Mechanical properties of neat PCL, PCL/PHA blend, and nanocomposite. The ratio of mechanical properties compared to the values for neat PCL are reported to highlight the difference between the blend and the nanocomposite.

Material	Young Modulus		Yield Stress		Stress at Break		Strain at Break		Toughness	
	(MPa)	Ratio	(MPa)	Ratio	(MPa)	Ratio	(%)	Ratio	(kJ/m^3^)	Ratio
Neat	330 ± 10	1.0	12.0 ± 0.4	1.0	24.0 ± 0.4	1.0	334 ± 10	1.0	40.4 ± 0.8	1.0
Blend	306 ± 15	0.9	11.4 ± 0.5	0.9	20.5 ± 0.4	0.8	357 ± 10	1.1	42.4 ± 1.2	1.0
Nanocomposite	690 ± 20	2.1	13.8 ± 0.4	1.1	43.7 ± 0.5	1.8	508 ± 15	1.5	80.5 ± 3.7	2.0

## Supplementary Materials

The following are available online at https://www.mdpi.com/2073-4360/12/11/2587/s1, Figure S1: Time-dependence of the tensile stress coefficient $\eta_{E}^{+}$ (t,$\dot{\varepsilon }$), for molten PCL, the PCL/PHA blend and in situ generated composite. Figure S2: Derivative weight loss (DTGA) of PHA, PCL, the PCL/PHA blend and in situ generated composite. Table S1: Peak (T_d_) temperatures of DTGA for neat PHA, PCL as well as PCL/PHA blend and composite during heating at 10 °C min^−1^ in nitrogen. Figure S3: The 2D WAXS of neat PCL, PCL/PHA blend and composite samples recorded at different elongation. The drawing direction is horizontal.
