# Supplementary material for: Microstructural Evolution of Poly(ε-Caprolactone), Its Immiscible Blend, and In Situ Generated Nanocomposites
Source: Polymers (Basel). 2020 Nov 4;12(11):2587. doi: 10.3390/polym12112587 (PMC7694185; doi:10.3390/polym12112587)
Supplement: Supplementary file 1 [file polymers-12-02587-s001.pdf]

Supplementary Materials: The following are available online at www.mdpi.com/xxx/s1,

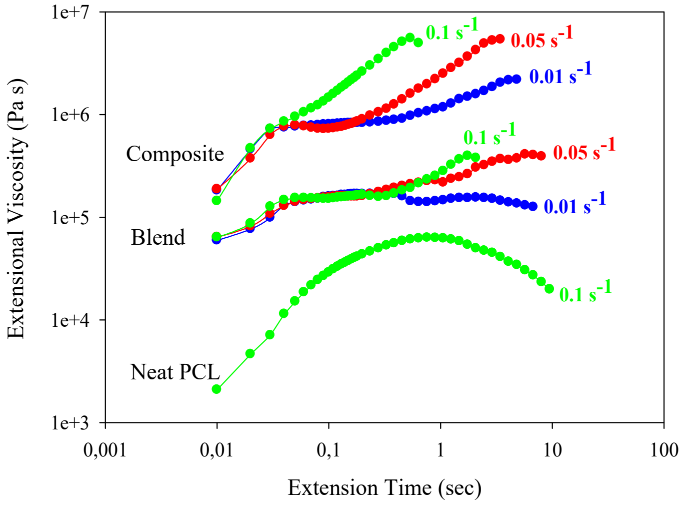

Fig.S1 Time-dependence of the tensile stress coefficient η_E^+ (t,$\dot{\varepsilon}$), for molten PCL, the PCL/PHA blend and in situ generated composite.

Fig.S1 presents a time-dependence of tensile stress growth coefficient η_E^+ (t,$\dot{\varepsilon}$) for molten PCL, PCL/PHA blend and nanocomposite recorded during uniaxial tension at various Hencky strain rates $\dot{\varepsilon}$. It is seen that neat PCL demonstrates a strain softening (decrease of η_E^+(t,$\dot{\varepsilon}$)) above linear viscoelastic region, while PCL/PHA blend and nanocomposite reveal strain hardening (an upward deviation of η_E^+ (t,$\dot{\varepsilon}$)). At the same time, strain hardening is more pronounced in the case of nanocomposite compared to blend which is associated with the presence of a larger number of load-bearing entanglements which are nanofibrils formed in the composite. With an increase in the strain rate, more strain hardening occurs, which can be attributed to the straining of the network of PHA entangled nanofibers.

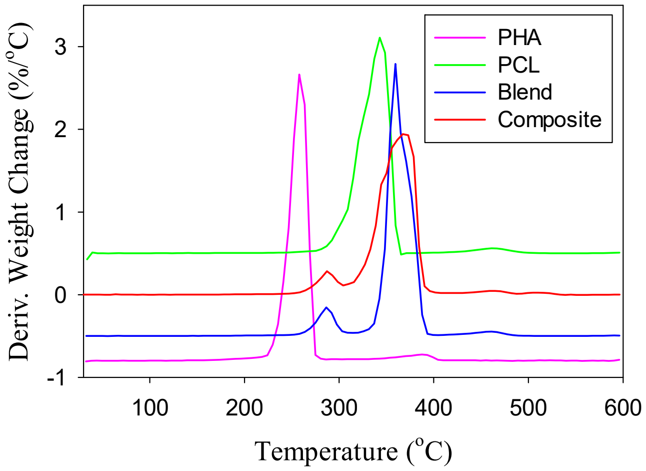

Fig.S2 Derivative weight loss (DTGA) of PHA, PCL, the PCL/PHA blend and in situ generated composite.

Derivative of weight loss (DTGA) of the materials during heating are plotted in Fig. S2, whereas peak (T_d) temperatures of DTGA plot are summarized in Table S1.

Table S1. Peak (T_d) temperatures of DTGA for neat PHA, PCL as well as PCL/PHA blend and composite during heating at 10 °C min^{-1} in nitrogen

Polymer	First peak	Second peak	Third peak	Fourth peak
	T_d, °C	T_d, °C	T_d, °C	T_d, °C
PHA	259	387	-	-
PCL	344	463	-	-
PCL/PHA blend	287	359	460	-
PCL/PHA composite	289	367	462	511

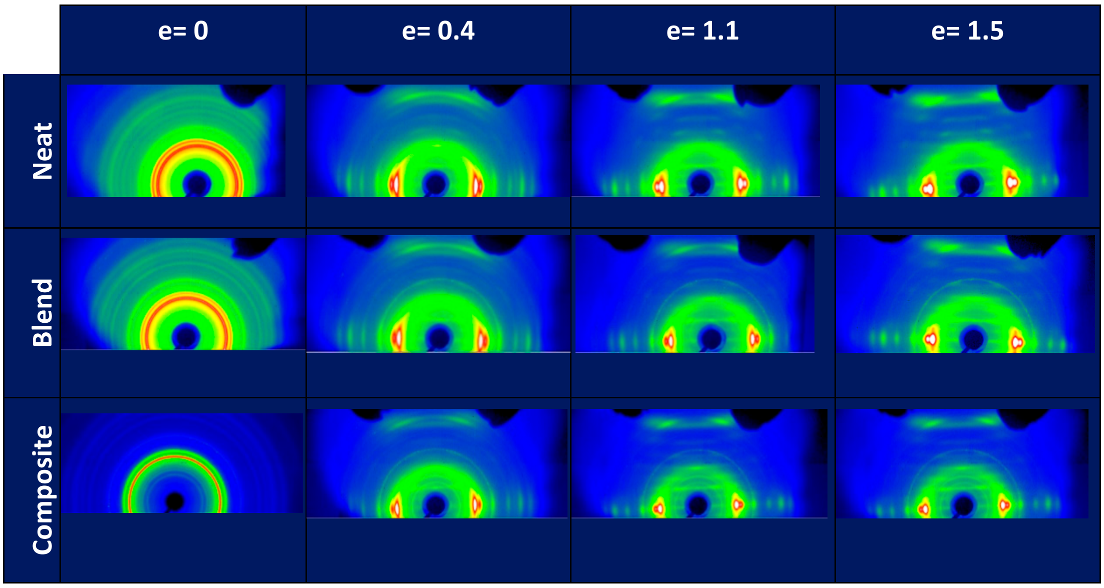

Fig. S3 The 2-D WAXS of neat PCL, PCL/PHA blend and composite samples recorded at different elongation. The drawing direction is horizontal.

Fig. S3 shows the representative 2-D WAXS patterns of neat PCL, PCL/PHA blend and composite for the range of true strain values 0 up to 1.5. The patterns were used to obtain the 110 reflection as a function of true strain and full width at half-maxima (FWHM).
